# Release of extracellular vesicles containing small RNAs from the eggs of *Schistosoma japonicum*

**DOI:** 10.1186/s13071-016-1845-2

**Published:** 2016-11-08

**Authors:** Shanli Zhu, Sai Wang, Yu Lin, Pengyue Jiang, Xiaobin Cui, Xinye Wang, Yuanbin Zhang, Weiqing Pan

**Affiliations:** 1 Institute for Infectious Diseases and Vaccine Development, Tongji University School of Medicine, Shanghai, China; 2 Department of Tropical Infectious Diseases, Second Military Medical University, Shanghai, China

**Keywords:** *Schistosoma japonicum*, Eggs, Extracellular vesicles, Small non-coding RNAs, miRNAs

## Abstract

**Background:**

*Schistosoma japonicum* is a parasitic flatworm that causes human schistosomiasis. Secreted extracellular vesicles (EVs) play a key role in pathogen-host interfaces. Previous studies have shown that *S. japonicum* adult worms can release microRNA (miRNA)-containing EVs, which can transfer their cargo to mammalian cells and regulate gene expression in recipient cells. Tissue-trapped eggs are generally considered the major contributor to the severe pathology of schistosomiasis; however, the interactions between the host and parasite in this critical stage remain largely unknown.

**Methods:**

The culture medium for *S. japonicum* eggs in vitro was used to isolate EVs. Transmission electron microscopy (TEM) analysis was used to confirm that vesicles produced by the eggs were EVs based on size and morphology. Total RNA extracted from EVs was analyzed by Solexa technology to determine the miRNA profile. The in vitro internalization of the EVs by mammalian cells was analyzed by confocal microscopy. The presence of EVs associated miRNAs in the primary hepatocytes of infected mice was determined by quantitative real-time PCR (qRT-PCR).

**Results:**

EVs were isolated from the culture medium of in vitro cultivated *S. japonicum* eggs. TEM analysis confirmed that nanosized vesicles were present in the culture medium. RNA-seq analysis showed that the egg-derived EVs contained small non-coding RNA (sncRNA) populations including miRNAs, suggesting a potential role in host manipulation. This study further showed that Hepa1-6, a murine liver cell line, internalized the purified EVs and their cargo miRNAs that were detectable in the primary hepatocytes of mice infected with *S. japonicum*.

**Conclusions:**

*Schistosoma japonicum* eggs can release miRNA-containing EVs, and the EVs can transfer their cargo to recipient cells in vitro. These results demonstrate the regulatory potential of *S. japonicum* egg EVs at the parasite-host interface.

**Electronic supplementary material:**

The online version of this article (doi:10.1186/s13071-016-1845-2) contains supplementary material, which is available to authorized users.

## Background

Schistosomiasis is a serious parasitic disease throughout the world’s tropical regions, affecting more than 230 million people according to conservative estimates [1]. Three main species of schistosomes infect humans, including *Schistosoma haematobium*, *Schistosoma mansoni* and *Schistosoma japonicum*. In China, *S. japonicum* remains a major public health problem, although considerable efforts have been devoted to controlling its transmission over the last few decades [2–4]. The complex life-cycle of schistosomes involves multiple developmental stages, including the egg, miracidium, cercaria, schistosomulum and adult worm. Tissue-trapped eggs are generally considered the major contributor to the severe pathology of schistosomiasis, because the parasite has developed strategies to both facilitate the establishment of infection and evade or manipulate the host immune response. Recent investigations have made some progress in the analysis of parasite-host interactions [5–8]. However, a deeper understanding of mechanisms of parasitism is urgently required for discovering novel interventions to control the disease.

Exosomes are a subtype of small (30–150 nm in diameter), membrane-enclosed vesicles, released by various types of mammalian cells in both normal and pathological conditions [9–11]. These vesicles originate from the inward budding of endosomal membranes, which creates multivesicular bodies that fuse with the plasma membrane to effect release [12, 13]. Originally assumed to be cellular waste products, exosomes are now considered highly bioactive extracellular vesicles (EVs) that facilitate intracellular communication [14]. The cargo of exosomes is complex and variable, containing bioactive proteins, functional mRNAs, miRNAs and other small non-coding RNA (sncRNA) species [9, 10, 15]. Therefore, these vesicles can “horizontally” transfer signals to neighboring cells and thus serve as mediators of intercellular communication [15–20]. The discovery of EVs in plants, fungi and bacteria drove the theory that exosome-mediated communication could be operated on a cross-species basis [21–23]. Recent studies of *Heligmosomoides polygyrus*, *Echinostoma caproni*, *Fasciola hepatica*, *Dicrocoelium dendriticum*, *Brugia malayi* and *Leishmania donovani* have demonstrated that EVs are secreted from parasites and may be taken up by host cells [24–29]. The vesicles produced by parasites can deliver virulence factors (proteins and non-coding RNAs) to the host, thus favoring pathogen survival and disease progression [24, 27, 29]. In addition, characterization of EVs released by *S. mansoni* schistosomula [30], *S. mansoni* adult worms [31] and *S. japonicum* adult worms [32, 33] suggested that EVs and their cargo could play a key role in parasite-host interplay. Based upon the increasing realization that EVs facilitate intercellular communication in eukaryotes, we speculate that they contribute to maintenance of the long-term host-parasite interactions during schistosomiasis.

Here, we show that the egg of *S. japonicum* releases EVs whose size and morphology are consistent with exosomes. These vesicles contain small RNAs, including parasite-specific miRNAs and conserved miRNAs that contain identical seed sites between the host and *S. japonicum*. Further studies showed that the secreted EVs are internalized by mammalian cells and their cargo miRNAs can be transferred to the recipient cells. Moreover, the egg EVs associated-miRNAs can be detected in the primary hepatocytes of mice infected with *S. japonicum*. These results suggest the regulatory potential of *S. japonicum* egg EVs at the parasite-host interface.

## Methods

### *Schistosoma japonicum* egg isolation, culture and culture medium collection

For collection of *S. japonicum* egg secretion products, New Zealand rabbits were percutaneously infected with approximately 1,200 *S. japonicum* cercariae that were shed from lab-infected snails (*Oncomelania hupensis*) obtained from the National Institute of Parasitic Disease, Chinese Center for Disease Control and Prevention. Eggs were isolated in sterile conditions from the livers of infected rabbits 6–7 weeks post-infection using the sieving and enzymatic method described by Cai et al. [34]. After collection, the freshly isolated eggs were thoroughly washed three times with PBS (pH 7.4) and then maintained in preheated RPMI-1640 culture medium (Life Technologies, Carlsbad, CA, USA) containing 100 U of penicillin, 100 μg/ml of streptomycin and 0.25 μg/ml amphotericin B (Life Technologies) at 37 °C under 5 % CO_2_ at a density of 1 × 10^4^ eggs/ml for 24 h. Following 24 h incubation, eggs and pellets were removed by centrifugation at 3,000× *g* at 4 °C. The culture medium was collected for further exosome isolation as described below.

### Exosome-like vesicle isolation

An ExoQuick-TC Exosome Precipitation Kit from System Bioscience (SBI, Mountain View, CA, USA) was used for exosome isolation according to the supplier’s protocols. In brief, the medium treated as described above was initially centrifuged at 3,000× *g* for 15 min to remove remaining eggs and cell debris. Exosome isolation reagents (0.2 volumes) were added to the supernatants and incubated at 4 °C overnight. The ExoQuick-TC/medium mixture was then centrifuged at 1,500× *g* for 30 min at room temperature. The supernatants were discarded and pellets were resuspended in 50 μl PBS and stored at -80 °C until further analysis.

### Transmission electron microscopy

For visualization of the vesicles, purified EVs were applied to 200 mesh formvar-coated EM grids (Agar Scientific, Essex, UK), incubated for 1 min at room temperature, and then excess sample was removed. The grids were stained with 1 % uranyl acetate (SBI) for 5 min. The grids were then loaded onto the sample holder of the JEM-1230 TEM (JEOL,Tokyo, Japan) and exposed to an 80 kV electron beam for image capture.

### Small RNA library preparation and analysis

For detection of RNA species, total RNA was extracted from EVs released by parasite eggs using Trizol (Life Technologies), and RNA quality was evaluated with an Agilent 2100 system (Agilent Technologies, Santa Clara, CA, USA). The small RNAs between 18 and 30 nt were isolated by 15 % polyacrylamide gel electrophoresis (PAGE) and then ligated into Illumina’s proprietary 5′ and 3′ adaptors, and further converted into single strand cDNA. The cDNA products were amplified using a 3′-adaptor reverse primer and 5′-adaptor forward primer (Illumina, San Diego, CA, USA). The purified PCR products were used for clustering and sequencing by an Illumina Genome Analyzer at the Beijing Genomics Institute, Shenzhen.

All unique sequences along with their associated read counts were determined from the raw data. First, low quality reads, 3′-adaptor null reads, insert null reads, 5′-adaptor contaminants and reads with polyA tails were filtered. The clean datasets were mapped to the draft *S. japonicum* genome sequences (sjr2_scaffold.fasta, downloaded from ftp://lifecenter.sgst.cn:2121/nucleotide/corenucleotide) using the Short Oligonucleotide Alignment Program (http://soap.genomics.org.cn). We investigated the length distribution of the perfectly matched small RNA reads in the libraries. By comparing our sequences with the non-coding RNAs collected in Rfam (Version 11.0) and the NCBI GenBank database, sequenced small RNAs were annotated to different categories, including rRNAs, small nuclear RNAs (snRNAs), repeat associated small RNAs, tRNAs and miRNAs. For miRNA analysis, the unmatched small RNAs were further analyzed against miRbase (version 21) and GenBank to identify known mature miRNAs. Finally, unannotated small RNAs were used for novel miRNA prediction analysis using Mireap (http://sourceforge.net/projects/mireap). RNAfold was used to predict hairpin-like structures.

### Quantitative real-time PCR (qRT-PCR) analysis of *S. japonicum* egg EV associated miRNAs

Stem-loop qRT-PCR was used to validate the presence of miRNAs in *S. japonicum* egg EVs. Total RNA of egg EVs, culture medium and residuum supernatants (collected after EV isolation) were extracted using Trizol. qRT-PCR analysis was performed as previously described [35]. Briefly, a stem-loop RT primer was used to reverse-transcribe mature miRNA to cDNA; the 10 μl reverse transcription reactions contained 150 ng of total RNA, 50 nM of each individual stem-loop RT primer, 0.5 mM dNTP (Takara, Dalian, China), 5 U M-MLV reverse transcriptase (Takara), and 2 U RNase inhibitor. The temperature program was 60 min at 42 °C, 15 min at 70 °C and then holding at 4 °C. qRT-PCR was performed using an Applied Biosystems (ABI, Carlsbad, CA, USA) 7500 Sequence Detection system. The 20 μl PCR reaction included 2 μl of RT product (1:1 dilution), 1 × SYBR Premix Ex *Taq* (Takara), 0.5 μM specific forward primer, and 0.5 μM common reverse primer. The reactions were performed at 95 °C for 30 s, followed by 40 cycles of 95 °C for 5 s and 60 °C for 34 s. Cel-miR-39 was used as an internal control, and the relative miRNAs level was calculated by the 2^-ΔΔCt^ method [36]. All reactions were run in triplicate. All primers used are listed in Table 1.

**Table 1:** Sequences of primers used for qRT-PCR

Gene	Name	Sequence (5′–3′)
sja-miR-71b	RT stem-loop primer	CTCAACTGGTGTCGTGGAGTCGGCAATTCAGTTGAGCGTCTCA
	Forward primer	ACACTCCAGCTGGGTGAAAGACTTGAGT
sja-bantam	RT stem-loop primer	CTCAACTGGTGTCGTGGAGTCGGCAATTCAGTTGAGACCAGCT
	Forward primer	ACACTCCAGCTGGGTGAGATCGCGATTA
cel-miR-39	RT stem-loop primer	CTCAACTGGTGTCGTGGAGTCGGCAATTCAGTTGAGCAAGCTG
	Forward primer	ACACTCCAGCTGGGTGTCACCGGGTGTAAAT
	Common reverse primer	CTGGTGTCGTGGAGTCGGCAA
mmu-U6	Forward primer	GCTTCGGCAGCACATATACTAAAAT
	Reverse primer	CGCTTCACGAATTTGCGTGTCAT

### Confocal analysis of *S. japonicum* egg EV uptake by murine liver cells

Murine liver Hepa1-6 cells were obtained from the ATCC (CRL-1830) and grown according to the standard protocol in Dulbecco’s modified Eagle’s medium (DMEM) (Life Technologies) supplemented with 10 % fetal bovine serum (Life Technologies). Hepa1-6 cells were seeded in 12-well plates (1 × 10^5^ cells/well) using advanced serum-free DMEM (Life Technologies) for 4 h. Purified EVs from *S. japonicum* eggs or Hepa1-6 cells were labeled with the green fluorescent dye PKH67 (Sigma-Aldrich, St. Louis., MO, USA) as described by Hazan-Halevy et al. [37] with minor modifications. Briefly, 10 μg of the PKH67-stained EVs were washed three times using a 300-kDa Amicon (Merck Millipore, Merck KGaA, Darmstadt, Germany) to remove excess dye, EVs were then added to the cells and incubated for 1 h at 37 °C. As a control for non-specific labeling of cells, PBS was stained with PKH67, washed, and added to the cells. Following 1 h incubation, the medium was aspirated, cells were washed twice with PBS, fixed with 4 % formaldehyde solution for 15 min, and washed twice more with PBS; nuclei were stained with 4′,6-diamidino-2-phenylindole (DAPI) (Life Technologies). Finally, the cells were observed using confocal fluorescence microscopy (Leica TCS SP5 II, Heidelberg, Germany).

### qRT-PCR analysis of *Schistosoma*-specific miRNAs

Hepa1-6 cells were incubated with EVs (10 μg total protein per well) derived from *S. japonicum* eggs or Hepa1-6 cell culture medium (negative control) for 20 h. After washing twice with PBS, the cells were then incubated with 0.25 % trypsin/EDTA (Life Technologies) for 3 min to eliminate non-specifically adhered EVs. Total RNA was extracted from the incubated cells using Trizol, and miRNAs were reverse transcribed into cDNA using Reverse Transcriptase M-MLV (Takara) as described above. Real-time PCR was performed to assess the *Schistosoma*-specific miRNAs in the treated Hepa1-6 cells. Cel-miR-39 was used as an internal control, and the relative miRNAs level was calculated by the 2^-ΔΔCt^ method.

### Isolation of primary mouse hepatocytes

To ascertain whether parasite-specific miRNAs were present in the primary hepatocytes of mice infected with *S. japonicum*, primary mouse hepatocytes were isolated by a two-step collagenase perfusion procedure, as described by He et al. [38] with minor modifications. Briefly, 6-week-old male C57BL/6 mice were purchased from the experimental animal center of the Second Military Medicine University and exposed percutaneously to 20 *S. japonicum* cercariae. Livers of *S. japonicum*-infected mice (49 days post-infection (dpi) and 80 dpi, *n* = 5, respectively) and uninfected mice were initially in situ digested with 0.04 % collagenase type IV and then further digested with 0.08 % collagenase type IV at 37 °C in a shaking bath for 30 min. The single cell suspensions were harvested by filtration through 400-mesh sieves for removal of the remaining tissue debris and parasite eggs. Next, hepatocytes were isolated by centrifugation of the resulting cell suspensions at 50× *g* for 4 min and further purified by centrifugation at 20× *g* for 4 min. Purified hepatocytes were resuspended in DMEM containing 20 μg/ml Ribonuclease A (Sigma-Aldrich) at 37 °C in a bath for 30 min to eliminate the miRNA released by schistosome eggs. After three times washing with PBS, the cell pellet was used immediately for extraction of total RNA or stored in liquid nitrogen until further analysis.

### qRT-PCR analysis of *S. japonicum* miRNAs in primary hepatocytes of infected mice

Total RNA of primary hepatocytes derived from infected and uninfected mice were extracted using Trizol, and parasite miRNAs were reverse transcribed into cDNA using reverse transcriptase M-MLV as described above. qRT-PCR analysis was performed to determine the presence of parasite-specific miRNAs in the primary hepatocytes of infected mice. The U6 gene was used as an internal control. The 2^-ΔCt^ method was used to calculate the level of miRNAs.

### Statistical analysis

Results were analyzed using SPSS software (version 16) and statistical analyses were performed using one-way ANOVA. A value of *P* < 0.05 was considered statistically significant. Data are expressed as mean ± standard deviation.

## Results

### Isolation and purification of schistosomal eggs

Rapid isolation of viable schistosomal eggs from host hepatic tissue in sterile conditions was a critical step for obtaining EVs. In this study, eggs were isolated and purified from infected rabbits using the sieving and enzymatic methodology described by Cai et al. [34]. The purified egg samples were examined under a light microscope, and we observed that most of the eggs contained a developing embryonic larva (Additional file 1: Figure S1).

### EVs isolation from schistosomal eggs and small RNA analysis

**Fig. 1:**
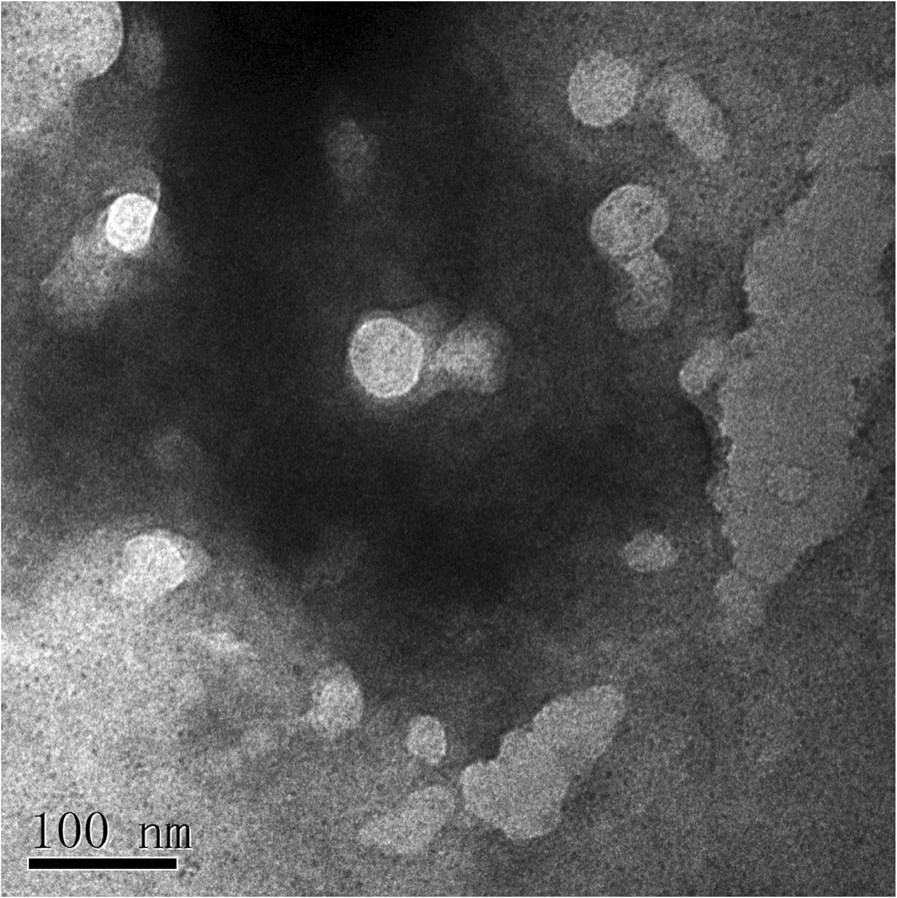
Characterization of extracellular vesicles (EVs) obtained from the eggs of *S. japonicum* by transmission electron microscopy (TEM). EVs were isolated from in vitro culture supernatants of *S. japonicum* eggs and analyzed by TEM at 200,000× magnification. *Scale-bar*: 100 nm

The schistosomal eggs were incubated for 24 h in standard culture conditions, and vesicles were purified from the culture medium and evaluated by TEM. TEM images showed that eggs cultivated in vitro secreted abundant 30–100 nm microvesicles consistent with the morphology previously described for *S. japonicum* adult worms [33], confirming that schistosomal eggs also release EVs (Fig. 1).

**Fig. 2:**
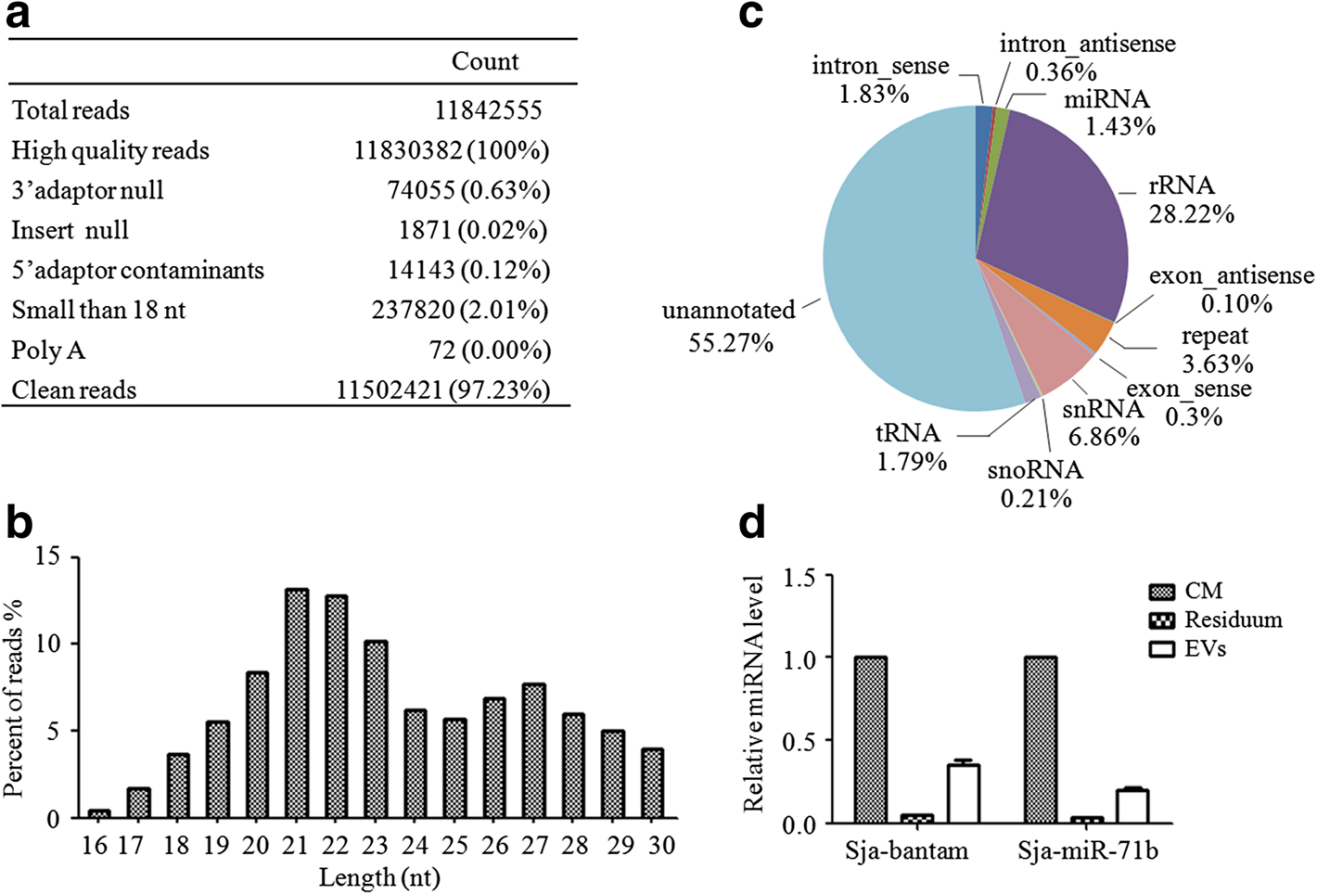
Identification of small RNAs associated with *S. japonicum* egg extracellular vesicles (EVs). **a** Summary of the output of the Solexa data; the percentage in parentheses indicates the percentage of high-quality reads. **b** The length distribution of small RNA tags. **c** Classification of the small RNAs by comparison with the *S. japonicum* genome. **d** qRT-PCR validation of the abundance of Sja-bantam and Sja-miR-71b in the RNA isolated from *S. japonicum* egg EVs. Values are normalized to culture medium, based on equal volumes of starting material. *Abbreviations*: CM, culture medium; EVs, extracellular vesicles; Residuum, supernatant medium from the final step of EV extraction

SncRNAs carried by EVs function as messengers for intercellular communication. Therefore, we identified the small RNA populations associated with schistosomal egg EVs by Solexa deep sequencing. As shown in Fig. 2a, b, the length distribution of schistosomal egg EV-associated small RNAs ranged from 18 to 30 nt. rRNAs, snRNAs and repeat associated small RNAs were the dominant classes of small RNAs, followed by intron_sense, tRNAs and miRNAs (Fig. 2c). We found 13 known *S. japonicum* miRNAs (reads >100) present in the schistosomal egg EV libraries (Table 2 and Additional file 2: Table S1), including three miRNAs (miR-10, bantam and miR-3479-3p) that were present in the plasma of *S. japonicum* infected host rabbits in a previous study [39]. In addition, one novel miRNA was predicted using Mireap (Table 2 and Additional file 2: Table S1). Then, stem-loop qRT-PCR was performed to verify the presence of Sja-bantam and Sja-miR-71b in the RNA isolated from schistosomal egg EVs (Fig. 2d). These findings demonstrated that schistosomal eggs release EVs during development in vitro and these 30–100 nm sized vesicles carry miRNAs that are both parasite-specific and homologs of mammalian (host) (e.g. mouse miR-10) miRNAs.

**Table 2:** List of identified miRNAs associated with *S. japonicum* egg EVs

Small RNA ID	Location				Sequence	Reads^a^	miRNA
t0000052	SJC_S000027	600247	600269	–	CCACCGGGTAGACATTCATTCGC	29608	sja-miR-36-3p
t0000207	SJC_S000052	314799	314820	+	AACCCTGTAGACCCGAGTTTGG	6156	sja-miR-10-3p
t0000525	SJC_S000254	288019	288040	+	TGAGATCGCGATTAAAGCTGGT	2307	sja-bantam
t0000729	SJC_S000054	245452	245472	–	TCACAGCCAGTATTGATGAAC	1332	sja-miR-2a-3p
t0000925	SJC_S000054	245576	245597	–	TGAAAGACGATGGTAGTGAGAT	1085	sja-miR-71a
t0001363	SJC_S000055	384663	384684	–	TATTGCACTTACCTTCGCCTTG	1070	sja-miR-3479-3p
t0001942	SJC_S000471	22294	22314	–	TATTATGCAACGTTTCACTCT	1038	sja-miR-2162-3p
t0001985	SJC_S000102	364277	364299	+	AAAGACTTGAGTAGTGAGACGCT	746	sja-miR-71b-3p
t0002533	SJC_S000054	245393	245413	–	CGTCTCAAAGGACTGTGAGCC	585	sja-miR-2b-3p
t0002630	SJC_S000664	24730	24752	+	TGACTAGAAAGTGCACTCACTTC	570	sja-miR-61
t0003175	SJC_S000001	925810	925830	–	TAAATGCATTTTCTGGCCCGT	554	sja-miR-277
t0003502	SJC_S004031	7481	7503	+	TCACAACCTACTTGATTGAGGGG	238	sja-miR-307
t0005635	SJC_S000102	364554	364577	+	TATCACAGTCCTGCTTAGGTGACG	139	sja-miR-2d-3p
t0007134	SJC_S000110	287436	287456	–	GGCCTCGTGGTGTAGCGGTTATC	105	novel-miR-7

^a^Only miRNAs with > 100 reads are listed

### Uptake of schistosomal egg EVs by murine cells

**Fig. 3:**
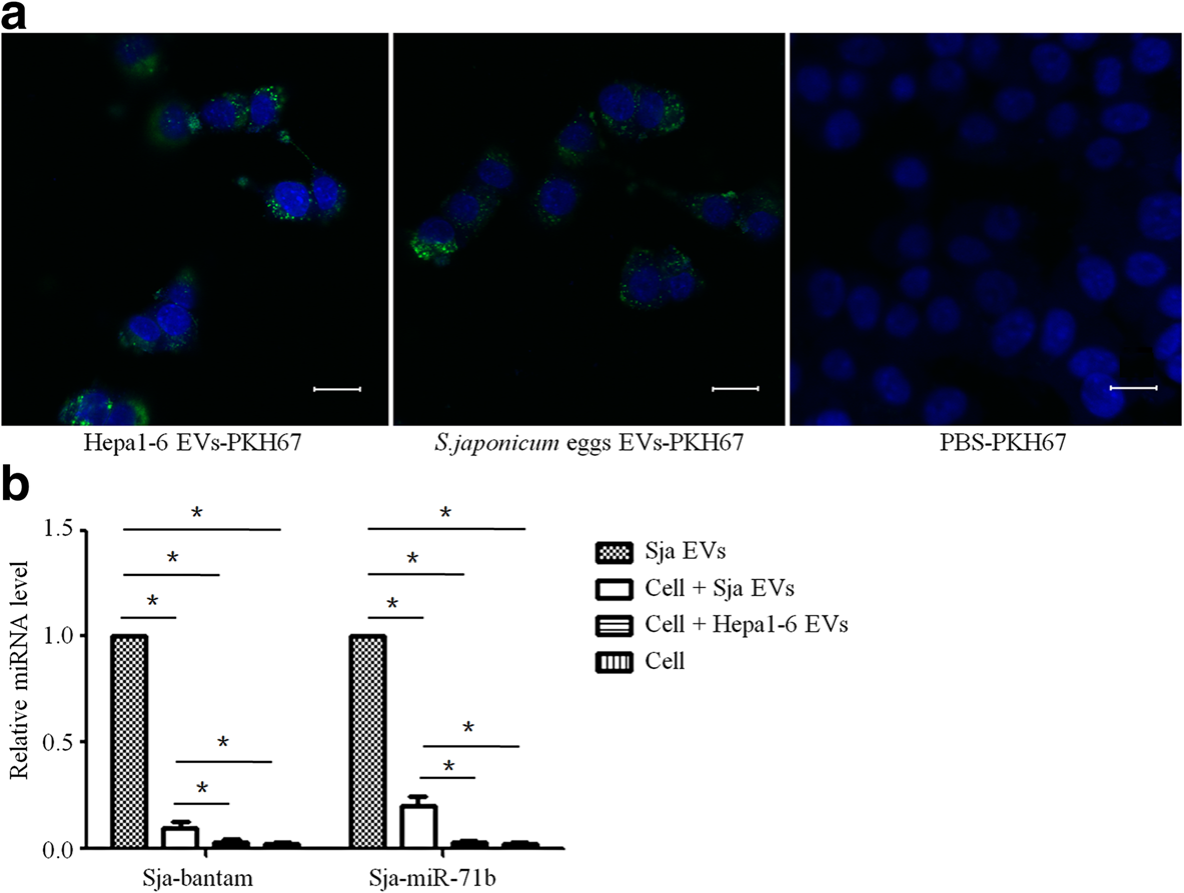
*Schistosoma japonicum* egg extracellular vesicles (EVs) and RNAs were internalized by murine liver cells. **a** Uptake analysis of *S. japonicum* egg EVs by mouse liver cells detected by confocal microscopy. PKH67-labeled *S. japonicum* egg EVs, Hepa1-6 EVs, and PHK67-PBS were incubated with Hepa1-6 cells for 1 h. Nuclei were stained with DAPI (*blue*). *Scale-bars*: 20 μm. **b** Relative level of parasite-derived miRNAs (i.e. bantam and miR-71b) in murine liver cells 20 h post-incubation with 10 μg *S. japonicum* egg EVs following PBS washing. The data were normalized to the level of miRNAs in 10 μg of EVs. *P ≤ 0.05

To ascertain whether the schistosomal egg-derived EVs can enter mammalian cells, internalization was examined in mouse liver cells. EVs originating from schistosomal eggs or Hepa1-6 cells (positive control) were labeled with the green fluorescent lipid dye PHK67 and incubated with Hepa1-6 cells in vitro. Confocal image analysis revealed efficient internalization of the parasite egg-EVs by murine Hepa1-6 cells (Fig. 3a). qRT-PCR analysis of the treated cells demonstrated that schistosomal egg EVs associated miRNAs (bantam and miR-71b) were detectable in Hepa1-6 cells after 20 h of incubation with parasite EVs (Fig. 3b). The above indicated that the miRNAs carried by schistosomal egg EVs can be transferred to recipient mammalian cells.

### qRT-PCR analysis of *S. japonicum* miRNAs in the primary hepatocytes of infected mice

**Fig. 4:**
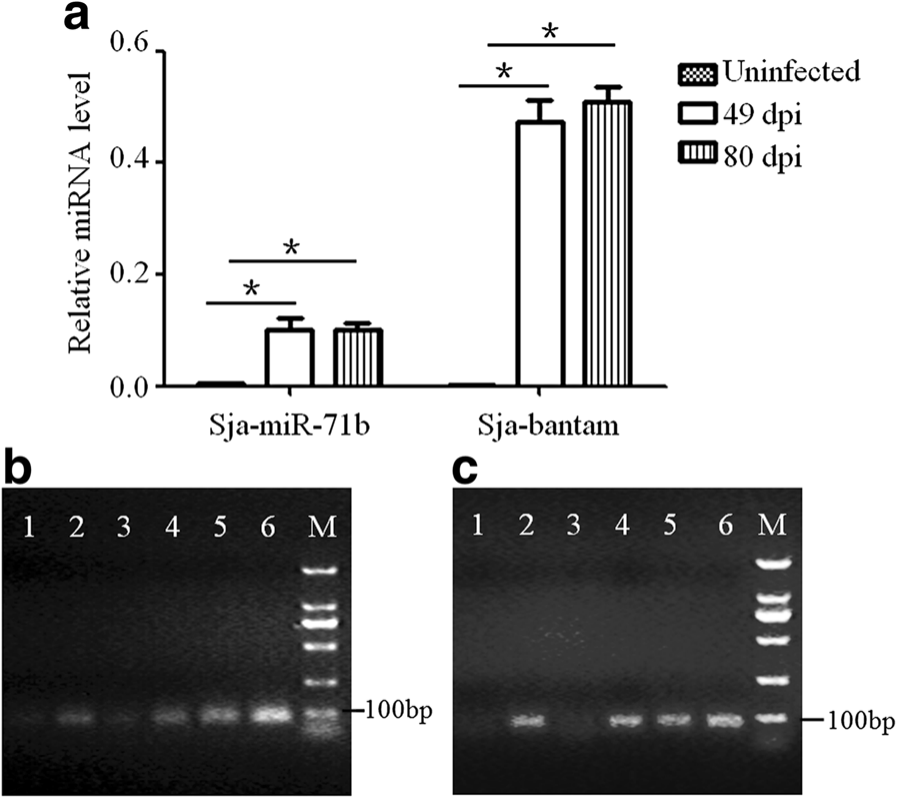
qRT-PCR analysis of the Sja-miR-71b and Sja-bantam level in primary hepatocytes of infected mice. **a** qRT-PCR analysis of two of the miRNAs that are associated with *S. japonicum* egg EVs (i.e. Sja-miR-71b and Sja-bantam) in primary hepatocytes of infected mice at 49 dpi and 80 dpi. **b**, **c** The PCR products of Sja-miR-71b (68 bp) and Sja-bantam (67 bp). Lanes 1 and 3: primary hepatocytes of uninfected mice at days 49 and 80; Lanes 2 and 4: primary hepatocytes of infected mice at 49 dpi and 80 dpi; Lane 5: *S. japonicum* egg EVs; Lane 6: *S. japonicum* eggs

To ascertain whether parasite-specific miRNAs were present in the primary hepatocytes of mice infected with *S. japonicum*, primary hepatocytes of infected mice were isolated and detected for two miRNAs that are associated with *S. japonicum* egg EVs (i.e. Sja-miR-71b and Sja-bantam). The result showed the two miRNAs were detectable in the primary hepatocytes at 49 dpi and 80 dpi (Fig. 4), indicating that parasite-derived miRNAs were present in the primary hepatocytes of infected mice after numerous eggs deposited in the liver.

## Discussion

Extracellular vesicles (EVs), and particularly exosomes, are emerging as one of the major mediators of intercellular communication within mammalian systems. EVs deliver their cargo molecules to specific cells and the transfer of molecules may modulate cellular activity and pathways in recipient cells. Recent studies revealed that helminth parasites such as *B. malayi*, nematode *H. polygyrus* adults, *S. mansoni* schistosomula and *S. japonicum* adults, can also release EVs enriched in proteins and non-coding RNAs [24, 27, 30, 33, 40]. However, it remained unknown whether *S. japonicum* eggs, the major agents causing the severe pathology of schistosomiasis, could release EVs. Here, we isolated and cultivated the tissue-trapped eggs of *S. japonicum*, and verified that in vitro cultivated eggs released nanosized vesicles into the culture supernatant (Fig. 1). These nanovesicles potentially represent novel modulators of host and parasite interactions, therefore, their sncRNA content was explored further.

RNA deep sequencing revealed that miRNAs were among the small RNAs in the schistosomal egg EV libraries. Considering RNAs with > 100 reads, 13 previously characterized miRNAs and one potential novel miRNA were identified. Among the 13 known Sja-miRNAs identified in the egg EVs, Sja-bantam, Sja-miR-10 and Sja-miR-3479-3p were all previously detected in serum obtained from rabbits infected with *S. japonicum* [39]. Also, it has been shown that Sja-miR-277 and Sja-miR-3479-3p were detectable in the serum samples of infected mice [41]. We speculate that the circulating miRNAs are protected from degradation by encapsulation in the EVs. Interestingly, bantam and miR-10 were significantly enriched in the libraries of EVs derived from schistosomal adult worms, whereas miR-3479-3p did not appear in those EV libraries [33]. Moreover, it has been shown that five miRNAs (miR-71, miR-71b, miR-1, miR-36, and miR-124) are the most abundant in the egg stage of *S. japonicum* [34], implying that these miRNAs play important roles in embryo development. In the present study, four of these miRNAs (all except Sja-miR-1) were incorporated into the egg EVs. A similar phenomenon was also observed in the adult stage of *S. japonicum* [33]. These findings support the notion that miRNAs are selectively packaged into exosomes and delivered to specific cells, although it remains unclear whether the sorting mechanism is related to association with the RNA-induced silencing complex components or the target mRNAs [42–44].

In mammals, cell-to-cell communication could be mediated by EVs, which can deliver their cargo miRNAs to recipient cells [13, 14]. Exosomal miRNAs have specific functions such as immune response activation and cell development [45, 46]. The helminthic nematode *H. polygyrus* utilizes exosomal vesicles to increase virulence in a fashion similar to that of the mammalian miRNA transport mechanism [24]. *H. polygyrus* secretes miRNA-loaded vesicles that are accompanied by a nematode AGO protein, most likely to stabilize the miRNAs. Remarkably, *H. polygyrus* vesicles are internalized by mouse cells, which results in suppression of host immunity. Some *H. polygyrus* miRNAs were shown to target in vitro host mRNAs that are related to host immunity. In addition, previous study has shown that exogenous plant miRNAs are not only present in serum and tissues of hosts after oral uptake, but they can also regulate the expression of specific genes in the host liver [21]. These findings support the notion of miRNA-mediated cross-kingdom regulation, although the mechanisms responsible for exogenous miRNA transfer and function remain largely unknown. In the present study, we showed that murine liver cells could internalize schistosomal egg EVs and their cargo miRNAs in vitro. The results were consistent with previous findings that EVs derived from nematodes and *S. japonicum* adults could be taken up by mouse small intestinal epithelial cells and liver cells, respectively [24, 33]. Therefore, EVs derived from *S. japonicum* eggs potentially act as vehicles to package and deliver miRNAs to host cells and regulate host gene expression, which may facilitate parasitism.

Several lines of evidence have shown that highly conserved miR-71 and bantam are packaged in parasite-derived EVs, including from *H. polygyrus*, *B. malayi* and *S. mansoni*, suggesting that conserved miR-71- and bantam-secretion systems might exist in helminths [24, 27, 30]. In *Drosophila*, bantam miRNA has been shown to target a tumor-suppress pathway, promoting cellular growth and suppressing cellular apoptosis [47]. Although the role of miR-71 secreted by parasites remains unclear, it has been proposed to be involved in host-pathogen interactions [24, 30]. In the present study, we observed that Sja-miR-71b and Sja-bantam are also incorporated into the EVs derived from schistosomal eggs and these miRNAs can be transferred to murine liver cells via EVs in vitro. More importantly, we found that the parasite-specific miR-71b and bantam were present in the primary hepatocytes of *S. japonicum* infected mice after numerous eggs deposited in the liver.

## Conclusions

*Schistosoma japonicum* eggs can release miRNA-containing EVs, which can transfer their cargo miRNAs to recipient cells in vitro. Moreover, egg EVs associated miRNAs (i.e. Sja-miR-71b and Sja-bantam) were detectable in the primary hepatocytes of mice infected *S. japonicum*. Further work is required to identify the protein components within *S. japonicum* egg EVs and to understand whether and how each of these cargo molecules contribute to the fundamental interactions between the parasite and its host. Further exploration of the functions of these vesicles may help identify novel strategies for control of schistosomiasis.

## Additional files

Additional file 1:
**Figure S1.** *S. japonicum* eggs isolated from rabbits infected with *S. japonicum* cercariae 44 dpi. (TIF 6650 kb)
Additional file 2:
**Table S1.** The list of schistosome small RNAs (over 100 reads) associated with *S. japonicum* egg extracellular vesicles (EVs). (XLSX 126 kb)

## Abbreviations

DAPI: 4′,6-diamidino-2-phenylindole
dpi: days post-infection
EVs: Extracellular vesicles
miRNAs: microRNAs
PAGE: Polyacrylamide gel electrophoresis
qRT-PCR: quantitative real-time PCR
sncRNA: small non-coding RNA
TEM: Transmission electron microscopy
