# Supplementary figures and images for: Release of extracellular vesicles containing small RNAs from the eggs of Schistosoma japonicum
Source: Parasit Vectors. 2016 Nov 8;9:574. doi: 10.1186/s13071-016-1845-2 (PMC5101684; doi:10.1186/s13071-016-1845-2)

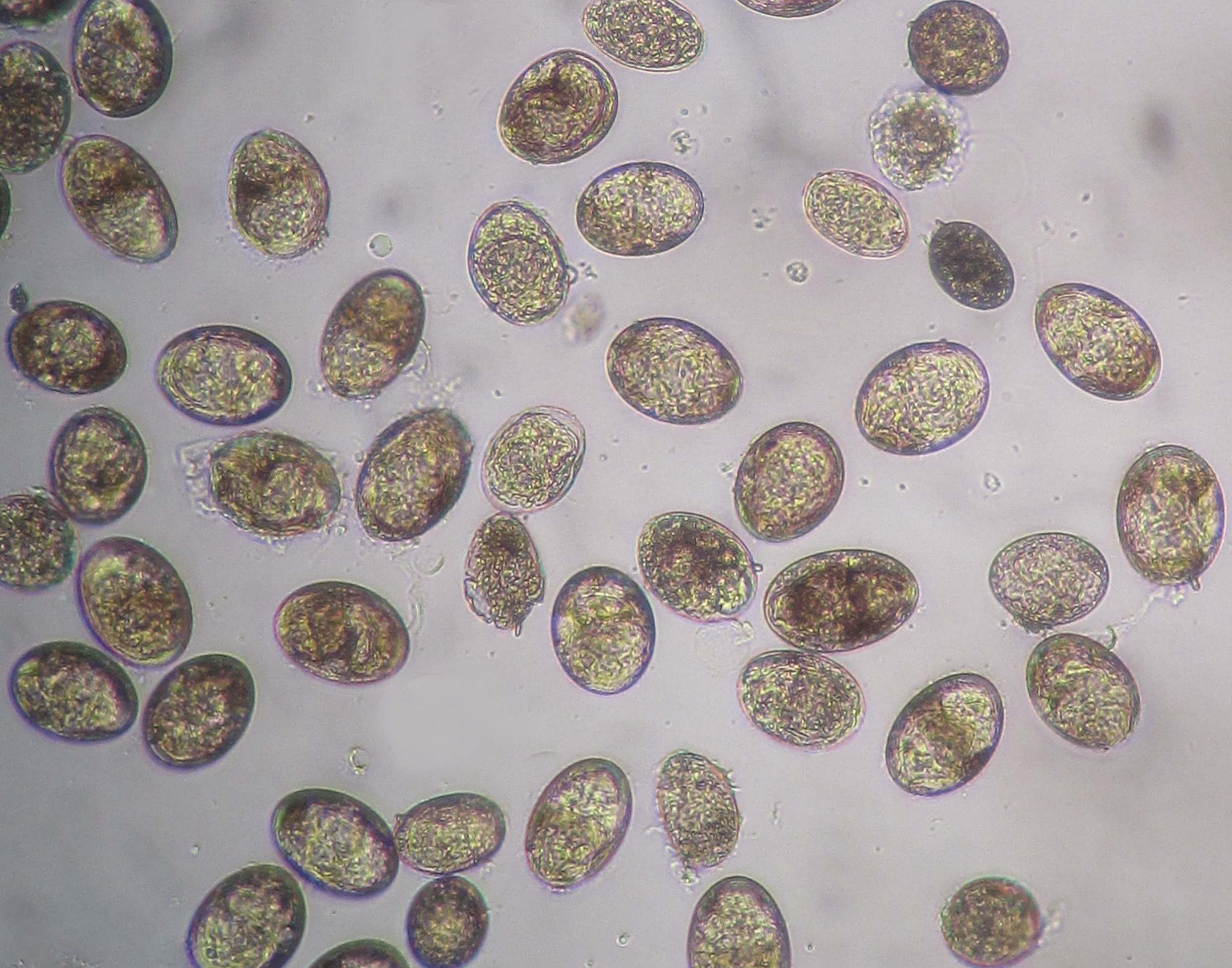

Supplement: Additional file 1: — Figure S1. S. japonicum eggs isolated from rabbits infected with S. japonicum cercariae 44 dpi. (TIF 6650 kb) [file 13071_2016_1845_MOESM1_ESM.tif]
